# Ascending colon injury and ileal perforation due to blunt abdominal trauma: A case report

**DOI:** 10.1016/j.radcr.2024.01.090

**Published:** 2024-02-17

**Authors:** Toshiyuki Suzuki, Daisuke Sugiki, Akiyo Matsumoto, Takahiko Akao, Hiroshi Matsumoto

**Affiliations:** a Department of Surgery, Hanyu General Hospital, Hanyushi Saitama, Japan; b Department of Emergency and Critical Care Medicine, Emergency and Critical Care Center, Dokkyo Medical University Saitama Medical Center, Koshigaya City, Japan

**Keywords:** Ascending colon injury, Ileal perforation, Road traffic accident, Seat belt syndrome, Blunt abdominal trauma

## Abstract

A rare case of an ascending colon injury and ileal perforation in a 34-year-old male patient due to blunt abdominal trauma caused by a road traffic accident is reported in this study. This paper reports the clinical and imaging findings of seat belt syndrome. The seat belt syndrome primarily involves soft tissue injury; however, lacerations of the colon, small intestine, and mesentery have rarely been reported in the literature. However intestinal injuries, including bowel perforation and mesenteric injuries due to seat belt syndrome, must not be underestimated because they usually require emergency laparotomy because of accompanying peritonitis and hemorrhaging, and can be lethal if left untreated. Therefore, when an ascending mesocolon hematoma and free gas in the peritoneal cavity are present, gastrointestinal perforation due to seat belt syndrome should be suspected. In this case, gastrointestinal perforation was suspected based on the computed tomography findings, and emergency surgery was performed; the patient's course was uneventful without any postoperative complications. Early diagnosis and management are essential to prevent associated morbidity and mortality.

## Introduction

Seat belt syndrome was first described by Garrett and Braunstein in 1962 and is defined as a group of hollow viscus and lumbar spine injuries associated with the use of seat belt restraints [1]. Denis et al. reported that, though in Canada a mandatory seat belt law has reduced the occurrence of fatal injuries, such as critical head and facial injuries and severe blunt abdominal trauma (BAT), the occurrence of intestinal injuries due to seat belt syndrome has increased [2]. Intestinal injuries are rare and occur in approximately 1.2% of motor vehicle occupant injuries [3]. However, intestinal injuries, such as intestinal perforations and mesenteric injuries, due to seat belt syndrome are usually accompanied by peritonitis and bleeding, requiring emergency laparotomy, which can lead to death if left untreated. Early diagnosis was performed using computed tomography (CT) or abdominal ultrasound, and emergency surgery was performed. Moreover, we could control intra-abdominal contamination caused by bleeding or perforation and increase survival rates. Therefore, these injuries should not be underestimated.

Here, we report a case of ascending colon injury and ileal perforation caused by seat belt syndrome due to BAT from a traffic accident, which was identified in CT examination.

## Case report

**Fig. 1:**
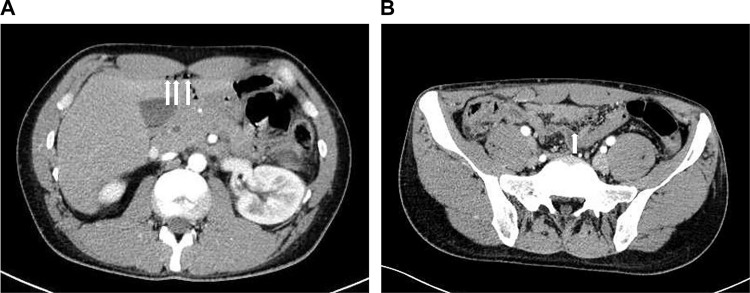
Pneumoperitoneum. (A) Axial section of CT of the abdomen showing free air on the surface of the liver (arrows). (B) Axial section of CT of the abdomen showing free air near the small intestine (arrows).

**Fig. 2:**
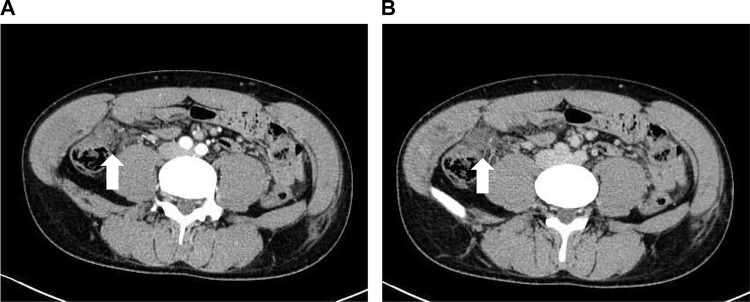
Mesenteric hematoma. Abdominal arterial (A) and portal (B) phase cross-sectional contrast-enhanced CT show a hematoma on the distal side of the ileocolic artery (arrows).

**Fig. 3:**
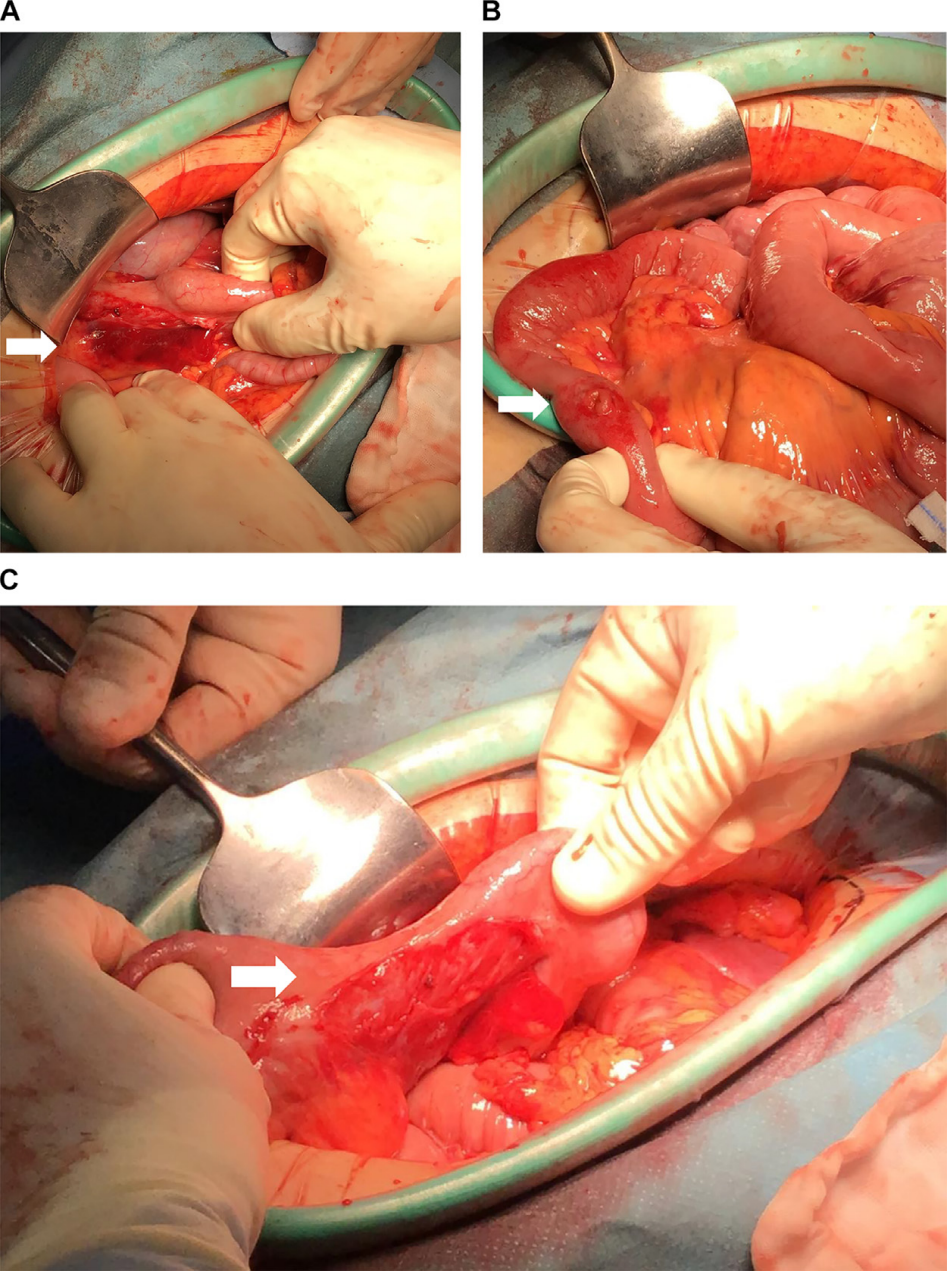
Intraoperative findings. (A) Surgical findings showing serosal injury of the ascending colon and a mesenteric hematoma (arrows). (B) Surgical findings showing ileal perforation (arrows). (C) Surgical findings showing serosal injury of the ascending colon (arrows).

**Fig. 4:**
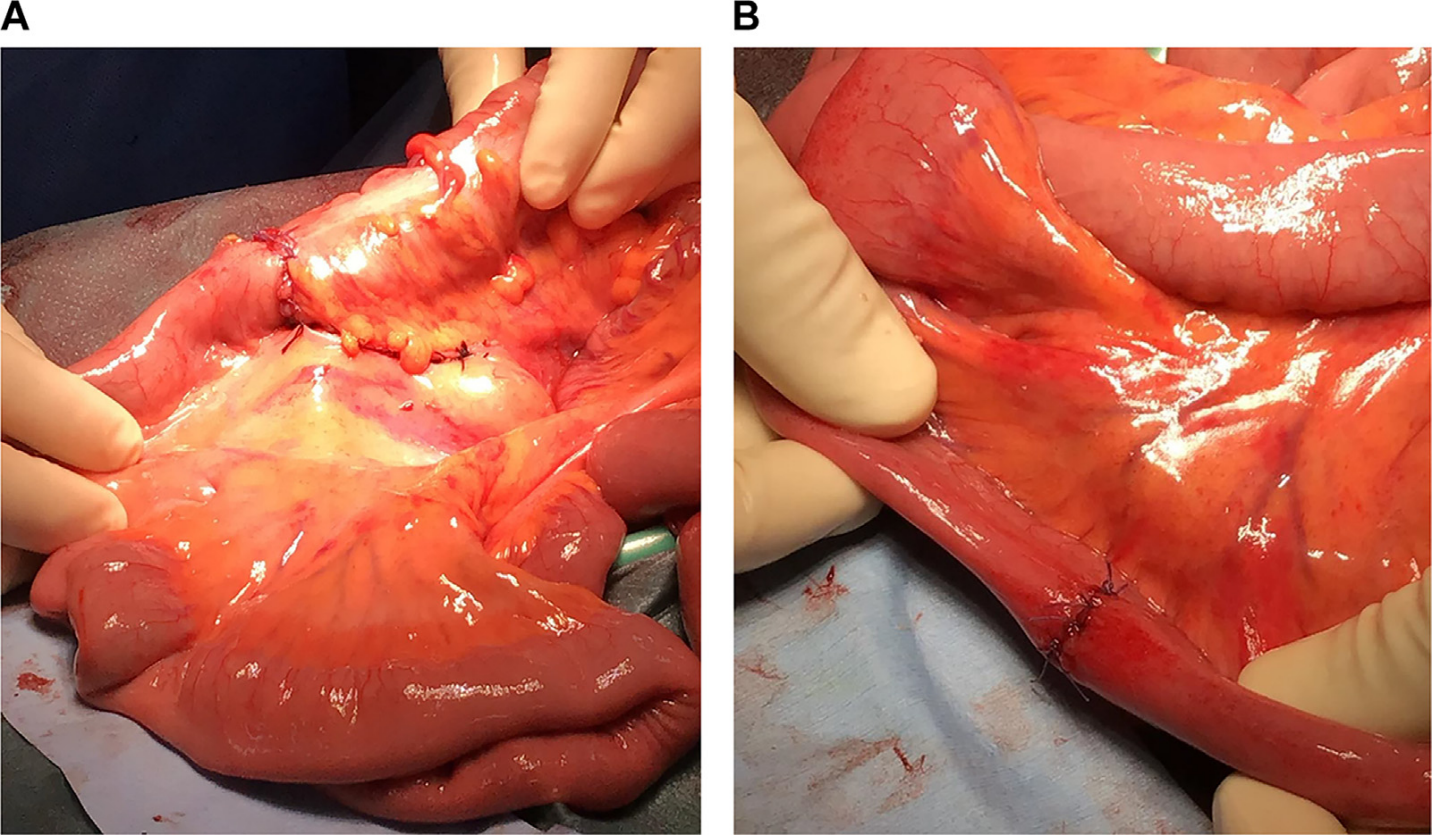
Completion of the surgery involving the colon and ileum. (A) Anastomosis and mesenteric closure after right hemicolectomy. (B) Simple closure of ileal perforation.

A 34-year-old male was brought to the emergency department by an ambulance after a traffic accident. An initial trauma assessment was performed. The patient was hemodynamically stable, with a Glasgow Coma Scale of 14/15, and no active external bleeding was observed. The patient complained of abdomen pain along the seat belt area and pain in the right clavicle area. Laboratory tests showed a white blood cell count of 13,980/µL. All other blood counts, electrolytes, urea, and other routine analyses were within normal ranges. Focused Assessment with Sonography for Trauma was negative. The initial differential diagnosis was abdominal contusion. Plain-film X-rays were taken and revealed right clavicle fractures. No pneumothorax or rib fractures were observed. An abdominal CT scan revealed free air on the surface of the liver and near the small intestine (Fig. 1). Furthermore, abdominal CT scan showed a hematoma in the mesentery near the ascending colon (Fig. 2). No obvious injuries to the liver parenchyma, spleen, pancreas, renal parenchyma, adrenals, ureters, major vessels, psoas muscles, or vertebral and pelvic bones were observed. Chest CT showed findings similar to those of plain-film X-rays. CT scans of the head and cervical spine did not reveal any abnormal findings. An exploratory laparotomy was performed to diagnose gastrointestinal perforation and ascending mesocolic hematoma. Intraoperative findings showed serosal injury of the ascending colon, mesenteric hematoma of the ascending colon, and ileal perforation (Fig. 3). No other obvious injuries within the adjacent parenchymal organs were observed. Right hemicolectomy and primary ileum repair were performed (Fig. 4). The patient made an uneventful recovery and was discharged after surgery for clavicle fracture. The abdominal pain was effectively controlled using painkillers.

## Discussion

Bowel injury in seat belt syndrome after BAT is rare; however, delayed diagnosis undoubtedly increases the occurrence of postoperative complications and, in the worst cases, leads to death. Therefore, knowing the characteristics of intestinal and mesenteric injury in seat belt syndrome after BAT and making an early diagnosis are important. Yamamoto et al. [4] reported that a small intestine was more vulnerable to perforation injury than a colon in 25 patients with seat belt syndrome. Dauterive et al. [5] and Watts et al. [6] also reported similar results to those of previous studies reporting BAT. In this case report, ileal perforation had similar results. Based on these results, for surgical procedures in a patient with bowel perforation, a surgeon should first inspect the small-bowel to quickly identify any injured sites. The characteristics of the perforated site of the small intestine have been reported for the entire small intestine and the jejunoileal junction; however, no definite opinion has been obtained. Regarding colon injury, some studies have reported that the ascending colon and sigmoid colon are common [4,5]. This case also showed damage to the ascending colon, consistent with previous reports. Regarding mesenteric injury, some studies have reported that the small mesentery is more common than the colon mesentery [4,5]. However, when the lesion is confined to the mesocolic ligament, the most common site is the ascending mesocolon [5]. In contrast, in this case, mesenteric injury of the small intestine was not observed. Mesenteric injury of the ascending colon was observed, consistent with previous reports. These differences in site distribution probably result from a unique mechanism of intestinal injury, involving direct compression and deceleration of the abdominal wall by the seat belt [7], [8], [9].

Ultrasonography can successfully assess free intraperitoneal fluid, particularly in patients with hemodynamic instability, but has little effect on the small pneumoperitoneum, as in this case. Therefore, CT is often the initial imaging modality used to assess patients with BAT. CT provides excellent anatomic details and diagnostic specificity by directly imaging the intestinal wall, detecting secondary signs of intestinal disease within the surrounding mesentery, and depicting even small amounts of extraluminal air into the peritoneal cavity [10,11]. Free intraperitoneal air is a specific sign of abdominal perforation. CT is the most reliable imaging modality for detecting even small amounts of free air [10,[12], [13], [14]]. CT findings suspecting small-bowel perforation include intestinal wall discontinuity, focal thickening of the intestinal wall adjacent to extraluminal air bubbles, and ascites [10,11,13,15,16]. Direct CT features that suggest perforation include extraluminal air, which is often associated with secondary CT signs of bowel pathology [17]. The overall accuracy in determining the perforation site is >80% [11]. In contrast, a study has reported that abdominal CT may not detect intestinal injury in 13% of cases [18]. Therefore, we believe that careful attention should be paid to CT findings after BAT. In particular, it is important not to overlook findings for free gas. If in doubt, CT reassessment after 2-3 h should be considered.

Additionally, it is important to be trained to interpret CT findings of gastrointestinal perforation. We identified the small and large intestines in CT images of strangulated intestinal obstruction and lower digestive perforation cases. For example, the entire large intestine from the anus to the cecum can be identified in a CT image. The identification of intestinal tract facilitates the identification of free gas and feces outside the intestinal tract. We held study sessions on CT interpretation with surgeons and radiology technicians regularly at our hospital. Therefore, early diagnosis could be made by identifying free gas around the small intestine and mesenteric damage in the large intestine.

The surgical procedure varies depending on intraoperative findings. Because small intestinal perforation was minor and no damage to the mesentery was observed, only primary ileum repair was selected for small intestinal perforation. Right hemicolectomy was chosen because of extensive damage to the colon (ie, serosal and mesenteric injuries).

Although automobile safety equipments, including seat belts, is highly developed, it is not possible to avoid traffic injuries. Therefore, seat bell syndrome occurs, and intestinal and mesenteric damage is difficult to prevent. Keeping in mind that seat belt syndrome can also cause damage to the intestines and mesentery may allow for early diagnosis and treatment, increasing the chance of survival.

## Conclusion

Although colon injury and ileal perforation due to seat belt syndrome are rare, this study showed that dangerous complications can be largely avoided if diagnosis is not delayed. CT offers unprecedented image processing power in providing a clear diagnosis compared to other imaging modalities. Signs of intraperitoneal free air strongly indicate gastrointestinal perforation. Therefore, accurately determining these signs is essential for maximizing the chances of an accurate diagnosis. Early diagnosis and management are important to prevent associated morbidity and mortality.

## Ethical statement

Not Applicable.

## Patient consent

Written informed consent for the publication of this case report and accompanying images was obtained from the patient. A copy of the written consent is available for review to the Editor-in-Chief of this journal on request.

## Author contributions

TS, DS, AM, TA, and HM contributed equally to this article as co-first authors. All authors have read the manuscript and agreed to its contents.

## Date availability

The datasets used and/or analyzed during the current study are available from the corresponding author upon reasonable request.
